# Effect of Different Temperatures on the Storage Stability of Flaxseed Milk

**DOI:** 10.3390/foods12193571

**Published:** 2023-09-26

**Authors:** Chen Meng, Yashu Chen, Xintian Wang, Hongjian Chen, Qianchun Deng

**Affiliations:** 1 School of Food and Biological Engineering, Hubei University of Technology, Wuhan 430068, China; m18407119868@163.com; 2 Key Laboratory of Oilseeds Processing, Ministry of Agriculture, Oil Crops Research Institute, Chinese Academy of Agricultural Sciences, Wuhan 430062, China; yashuchen@sina.com (Y.C.); wxin_t@163.com (X.W.); 3 College of Health Science and Engineering, Hubei University, Wuhan 430062, China

**Keywords:** flaxseed, plant-based milk substitutes, oxidative stability, physical stability

## Abstract

In this study, the physical and oxidative stability of flaxseed milk without food additives at different temperatures (25 °C and 37 °C) was assessed. Over in 206 days in storage, the particle size, Turbiscan stability index (TSI), centrifugal sedimentation rate, and primary and secondary oxidation products of flaxseed milk increased, viscosity decreased, and the absolute value of the potential first decreased and then increased. These phenomena indicated a gradual decrease in the physical stability of flaxseed milk, accompanied by drastic oxidative changes. The antioxidant capacity of flaxseed milk was related to the location of the physical distribution of flaxseed lignin, which was more effective in the aqueous phase compared to the non-aqueous phase. Interestingly, after 171 days in storage at 37 °C, the particle size of flaxseed milk was approximately doubled (6.98 μm → 15.27 μm) and the absolute value of the potential reached its lowest point (−13.49 mV), when the content of primary oxidation products reached its maximum (8.29 mmol/kg oil). The results showed that temperature had a significant effect on the stability of flaxseed milk and that stability decreased with increasing temperature and shortened shelf life. This work provides a theoretical basis for elucidating the stabilization–destabilization mechanism of flaxseed milk.

## 1. Introduction

With the continuous upgrading of food production technology and the increasing health and environmental awareness of consumers, plant-based foods are thriving [1]. Plant-based milk substitutes are new products developed in response to this trend, as there is growing interest due to its low saturated fat, low cholesterol content, and high dietary fiber [2].

Plant-based milk substitutes have been reported to play an important role in improving or managing the immune system [3,4,5]. They may also reduce the risk of low bone mass and have high levels of antioxidants while improving physiological function and possessing free radical scavenging properties, etc. [6]. These alternatives are also referred to as non-dairy alternatives, which mainly include soya, almond, peanut, walnut, and coconut milk. Various other sources of crops are also used to produce plant-based milk, but in relatively small quantities, such as flaxseed.

Flaxseed (*Linum usitatissimum* L.), also known as caraway seed, is one of the major oil crops in the word and is considered to be a major functional food [7]. Research has confirmed the significant role of flaxseed in the prevention and treatment of cardiovascular disease, regulation of human estrogen levels, and enhancement of intestinal health [4,8]. The plant-based milk substitute made from flaxseed not only has excellent nutritional and health characteristics by integrating the macro- and micronutrients of flaxseed but can also be green and additive-free with low food safety risks. Plant-based foods are designed to meet the needs of the general public for a new era of healthy products. As a new milk alternative, they address the disadvantages of animal protein drinks that are high in saturated fatty acids and cholesterol; however, some protein and lipid deficiencies and environmental factors make plant-based milk substitutes much less physically stable than milk [9]. Jeske et al. found that most plant-based milk substitutes have separation rates 14 times quicker than milk [10]. The systems of plant-based milk substitutes are complex in that they contain emulsions and other particles, including residual plant cells, soluble and insoluble proteins, dietary fiber, lipids, and carbohydrates [9]. These components may interact with each other within the system in suspension. During storage, plant-based milk substitutes are prone to stratification, flocculation, and sedimentation, which can affect the quality and sensory aspects of the product. Plant-based milk substitutes as a colloid may exist in the form of an emulsion or suspension. The stability of plant-based milk substitutes are influenced by various factors. These factors include the characteristics of the colloidal particles present, such as their size, density, surface charge, interfacial chemistry, and surface hydrophobicity. The nature of the surrounding aqueous phase, including its pH, ionic strength, composition, density, and viscosity, also plays a role. Additionally, the stability of the product can be influenced by environmental conditions during its service life, such as temperature changes, storage duration, and mechanical forces. Previous attempts have been made to explain the interactions within colloidal systems using the double-layer theory and DLVO theory, but hydration and hydrophobic and steric interactions may also exist in plant-based milk substitutes [11,12]. Electrostatic interactions may also arise from interactions between some components, and ζ-potential values can be indicative of the electrostatic behavior of particles in colloids [13]. Flaxseed milk is enriched with n-3 polyunsaturated fatty acids, which are prone to oxidative mass transfer triggered by the oil core, thus changing interfacial composition and charge distribution. This further alters the balance between electrostatic repulsion and van der Waals gravitational forces on the surface of the oil droplets, which affects the stability of flaxseed milk. Flaxseed milk with no food additives has a strong relationship to stability from its physical state, chemical oxidation and decomposition. 

In the current work, whole flaxseed was processed and used to prepare a flaxseed milk without food additives. The study explored the changes in the physicochemical stability of flaxseed milk at different temperatures and the interfacial distribution of the main antioxidant substances in flaxseed milk. This work may serve as a theoretical foundation for clarifying the stabilization–destabilization mechanism and provide theoretical support for expanding the market applications of flaxseed milk. It is also expected to be a reference for elaborating on and improving the storage stability of plant-based milk substitutes.

## 2. Materials and Methods

### 2.1. Materials and Chemicals

Flaxseed was purchased from Gansu Academy of Agricultural Sciences (Lanzhou, China). 2-thiobarbituric acid (TBA), 1,1,3,3-tetraethoxypropane, sodium azide, and cumene hydroperoxide were purchased from Sigma-Aldrich (Sigma-Aldrich, Saint Louis, MS, USA). All other reagents were acquired from Sinopharm Chemical Reagent Co., Ltd. (Sinopharm Chemical Reagent Co., Ltd., Shanghai, China).

### 2.2. Flaxseed Milk Preparation

A 9.5 cm-diameter Petri dish filled with flaxseed (19 g) was placed in a microwave oven (CEM Mars-6, North Carolina, USA) at 700 W for 5 min [14]. The microwave-treated flaxseeds were degummed in an aqueous solution at 40 °C (1:10, *w*/*v*) for 2 h. The flaxseeds were vacuum-precooled at 4 °C with deionized water (1:8, *w*/*v*; pH = 7.0) and ground (Horizontal-60, Shenzhen, China). The resulting emulsion was hydrolyzed with 1% cellulase and 2% glucoamylase for 1 h at 50 °C. The emulsion was filtered through a 200 mesh filter cloth and collected. High-pressure homogenization was performed after inactivation of the enzyme at 95 °C for 15 s (GYB40-10S, Shanghai, China). Finally, after UHT sterilization at 137 °C for 15 s, the flaxseed milk was stored for further analysis (ST21-4338-1, Shanghai, China).

### 2.3. Storage Experiment

Two quantities of flaxseed milk were stored in the dark at 25 °C and 37 °C, respectively, for 206 days. At each sample point, three bottles of flaxseed milk were tested. The flaxseed milk stored at a temperature of 25 °C was sampled every two weeks, resulting in a total of six samples. Additionally, the flaxseed milk stored at 37 °C was sampled once a week, yielding a total of ten samples. 

### 2.4. Measurement of Particle Size and ζ-Potential

The mean particle size and size distribution of the flaxseed milk were measured via static light scattering (Mastersizer 2000, Malvern Instruments, Westborough, MA). The refractive indexes of flaxseed oil and water phases were 1.490 and 1.330, respectively, which were used in the calculation of particle size. The ζ-potential of emulsions were determined via particle electrophoresis instrument (ZetaSizer-Pro, Malvern Instruments, Worcestershire, UK). The flaxseed milk samples were diluted with buffer solution (*v*/*v*, 1:250).

### 2.5. Determination of Viscosity

The static experimental samples were cooled to 25 °C, and the viscosity of the samples was measured with a DV-II digital viscometer (DV-II, Brookfield, MA, USA). After the corresponding rotor and speed were selected, the amount of liquid was taken by the diffuse viscometer rotor.

### 2.6. Evaluation of Gravity Separation Stability

A Turbiscan Lab Expert stability analyzer (Formulaction Inc., Toulouse, France) was used to monitor the change to the overall instability of the emulsion over time, to clarify the gravity separation stability of the emulsion, and to provide a basis for further evaluation of physical stability during storage. Turbiscan software determined the Turbiscan stability index (TSI).

After the emulsion was processed for the corresponding time, it was shaken well, and 10 g of the sample was accurately weighed and centrifuged at 1900× *g* (J-25, Beckman Coulter, CA, USA) for 15 min. The precipitate weight at the bottom of the centrifuge tube was recorded to calculate the centrifugal precipitation rate:

$$Centrifugal\;precipitation\;rate\left(\%\right)=\frac{Precipitate\;weight\;(g)}{Centrifugal\;sample\;weight\;(g)}\times 100$$

### 2.7. Observation of Microstructure

The microstructure of emulsions was observed via confocal laser microscopy (CLSM) (Nikon D-Eclipse C1 80i, Nikon, Melville, New York). A 20 μL quantity of Nile red-ethanol solution (1 mg/mL) was added to 200 μL of emulsion and buffer (*v*/*v*, 1:1) mixed solution [15]. The excitation and emission wavelengths of 543 nm and 605 nm were used to record the oil phase images (NISElements, Nikon, Melville, NY, USA).

A conductive carbon gel was applied to the sample stage, and the emulsion was dripped onto the conductive carbon gel. The sample stage with the sample adhered to it was snap-frozen in liquid nitrogen slush and transferred to the sample preparation chamber for sublimation and gold plating under vacuum using a cryogenic frozen preparation transfer system (PP3000T, Quorum, East Sussex, UK). Samples were sublimated at −90 °C for 10 min, followed by sputtering gold-plating at 10 mA for 60 s, then were sent to the scanning electron microscopy (SEM) sample chamber for observation (Quanta 450, FEI, OR, USA) (cold stage temperature −140 °C, accelerating voltage 5 kV).

### 2.8. Determination of Lipid Hydroperoxide

The primary oxidation product in the emulsion was mainly lipid hydroperoxide. The determination method is described by Kiralan [16]. A 0.3 mL quantity of flaxseed milk was mixed with 1.5 mL isooctane/isopropanol (*v*/*v*, 3:1), and the emulsion was destroyed by vortex. Then, the mixed solution was centrifuged at 2968× *g* for 10 min to obtain the supernatant. The upper solution (200 μL) was mixed with 2.8 mL methanol / butanol (*v*/*v*, 2:1), 15 μL 3.94 M ammonium thiocyanate and 15 μL Fe^2 +^ were added. The Fe^2+^ solution was prepared from an equal amount of 0.132 M BaCl_2_ and 0.144 M FeSO_4_. The 200 uL supernatant was replaced with isooctane/isopropanol (*v*/*v*, 3:1) as blank control. The above mixed solution was vortexed and incubated at room temperature for 20 min. After the reaction was completed, it was measured at 510 nm with a spectrophotometer (DU 800, Beckman Coulter, CA, USA). The standard curve was made with cumene hydroperoxide as standard to determine the concentration of hydrogen peroxide. All values are expressed as mmol cumene hydroperoxide equivalent per kilogram of oil.

### 2.9. Determination of Lipid TBARS

The determination method of secondary reaction products of lipid oxidation was described by Liang [17]. A 1 mL quantity of flaxseed milk was mixed with 2 mL of TBA reagent, destroyed by vortex. Then, it was placed in boiling water above 90 °C for 15 min and cooled to room temperature in a cold water bath. The supernatant was centrifuged at 2968× *g* for 15 min, which was measured for absorbance at 532 nm. The TBARS concentration was calculated from the standard curve of 1,1,3,3-tetraethoxypropane (TEP) to be mmol TEP equivalent per kilogram of oil. The TBARS solution was prepared according to the method previously described by Cheng et al. [15].

### 2.10. Distribution of Flaxseed Lignans in Flaxseed Milk

The physical location of flaxseed lignins in the emulsion was determined according to the method described by Panya et al. [18] with some modifications. A 1 mL quantity of emulsion was centrifuged at 5180× *g* for 1 h at 4 °C using a high-speed centrifuge. The flaxseed milk was divided into three layers, and the aqueous phase was carefully collected using a needle and syringe. Afterward, 1 mL of methanol was added to the remaining portion and vortexed vigorously. The supernatant was obtained after centrifugation and carefully collected using a needle and syringe, as above. The concentration of flaxseed lignans in the aqueous and non-aqueous phases was determined via HPLC (Waters, MA, USA) using an ACQUITY UPLC^®^ BEH Shield RP 18 column (2.1 × 100 mm, 1.7 μm). The solvents were 100% methanol and 0.5% acetic acid solution, and the injection volume was 2 μL. The non-linear gradient elution was performed at an elution flow rate of 0.1 mL/min. The detector wavelength was set to observe the sample peak at 280 nm. Flaxseed lignan macromolecules (purity > 95%) were used as the standard to configure the concentration gradient using 70% methanol water, and the sample was injected according to the above method. The standard curve was plotted for quantification with R^2^ > 0.999.

### 2.11. Statistical Analysis

All test results were taken as the average of three parallel measurements, which were presented as mean ± standard deviations. The statistical significance of the results was determined via analysis of variance (ANOVA) and the Tukey test, using SPSS 21.0 software (SPSS, Inc., Chicago, IL, USA). A threshold of *p* < 0.05 was employed to determine statistical significance.

## 3. Results and Discussion

Over 150 days in storage, the flaxseed milk underwent relatively little change in the indicators, so the data after 150 days was selected for graphical analysis.

### 3.1. Particle Size and ζ-Potential

**Figure 1:**
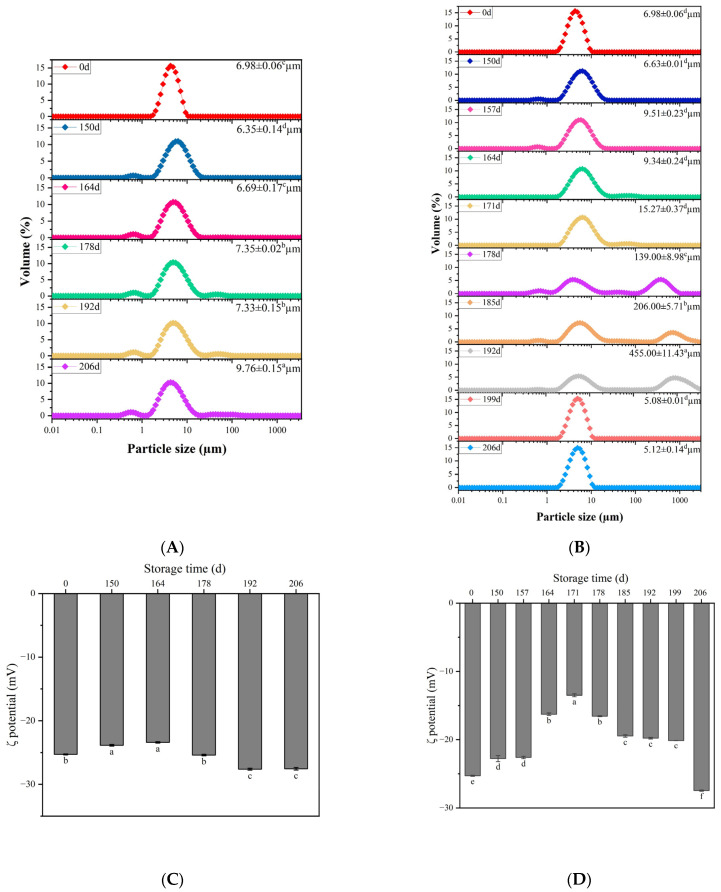
Particle size distribution of flaxseed milk at 25 °C (**A**) and 37 °C (**B**) at 206 days in storage. ζ-potential of flaxseed milk at 25 °C (**C**) 37 °C (**D**) at 206 days in storage. Different letters following the mean in the same column indicate significant differences (*p* < 0.05).

The variations in particle size distribution and D_4,3_ of flaxseed milk during storage are presented in Figure 1A,B. Fresh flaxseed milk (day 0) showed a moderately narrow single-peak distribution with a D_4,3_ of 6.98 μm. The particle size distribution of flaxseed milk changed from initially narrow to a broad single peak, with a small peak at 0.1 μm to 1 μm, after 164 days in storage at 25 °C. However, after 178 days, there was another small peak at 10 μm to 100 μm, which indicated particle flocculation or aggregation. At 178 days in storage at 37 °C, the flaxseed milk showed a large peak at 100 μm to 1000 μm. As reported by other researchers, the 0.1–10 μm peak might represent protein particles and oil bodies [19]. Oil bodies consist of surface proteins surrounding oil droplets, which stabilize flaxseed milk [20]. The intermediate particle size peaks of 10–100 μm might have included oil droplets and protein aggregates [19,21]. Maximum particle size peaks between 100 and 1000 μm might have consisted of aggregates of oil droplets or particles. Some plant-based milk substitutes, such as almond, cashew, oat, quinoa and rice beverages, present themselves as a polydisperse system [10]. Their suspension system contains a diverse range of interactions due to the presence of both large and small particles, including residual decomposed plant cells, soluble and insoluble proteins, lipids, and carbohydrates [22]. With increasing storage time at 37 °C, various interactions might have occurred between the various components of flaxseed milk, such as proteins, polysaccharides (flaxseed gum), and polyphenols (flaxseed lignans). At 199 days, the flaxseed milk showed a single peak distribution, presumably breaking the stable three-dimensional gel network structure formed by flaxseed milk and causing the originally stable large particles complex to break up into smaller particles.

The ζ-potential of the droplet affects the physical stability and the oxidative stability of flaxseed milk [23]. The variation in potential of flaxseed milk during storage is shown in Figure 1C,D. The flaxseed milk that was stored at 25 °C for 206 days showed an increase in potential to 2.21 mV. This suggests that flaxseed milk was exposed to some lipid droplet and interfacial oxidation during storage, during which oxidation of lipids or anionic reactions formed via hydrolysis might have led to a change in ζ-potential [24]. The absolute values of the potentials of the flaxseed milk stored at 37 °C increased after decreasing to the lowest point after 171 days in storage. This might be due to the aggregation of large particles weakening the electrostatic interaction between the particles. Generation of more small particles increased the specific surface area of the particles, which resulted in an increase of negative charge, causing a jump in potential values in the later stages. This was consistent with the particle size distribution results.

### 3.2. Visual Appearance and Microstructure

**Figure 2:**
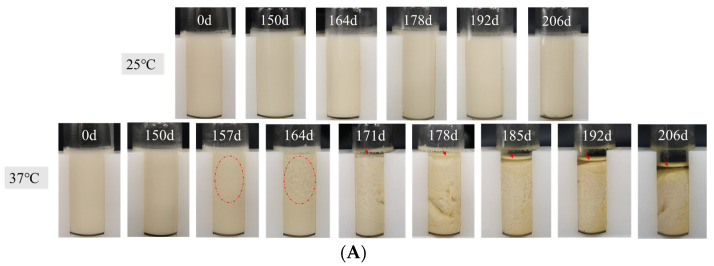

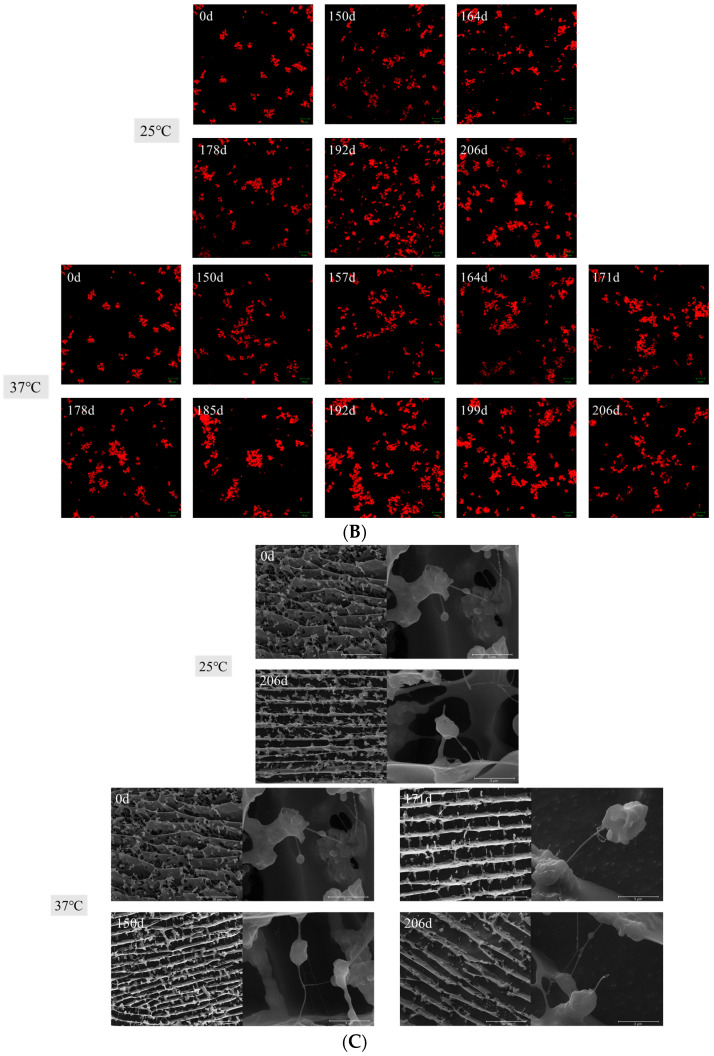
The visual appearance of flaxseed milk during storage at different temperatures for 0–206 days (**A**) and CLSM plots and (**B**) cryo-SEM plots (**C**). The scale bar in (**B**) is 10 μm.

The overall appearance of flaxseed plant-based milk at different temperatures during storage was shown in Figure 2A. Originally, the flaxseed milk had a homogeneous, cream-like appearance, indicating a uniform distribution of oil droplets. The color of the flaxseed milk was bright and homogeneous, mainly presenting as creamy and slightly yellow. There was little overall change to the color of flaxseed milk stored at 25 °C for 206 days. The flaxseed milk appeared to aggregate after 157 days in storage at 37 °C, darkened in color by 178 days, and oiling-off occurred at 185 days. A slight amount of oil phase precipitated from the upper layer of the flaxseed milk at 171 days in storage. The flaxseed milk gradually decreased in liquid level between days 178 and 220 in storage. Throughout storage, the interfaces between the flocculated particles and the continuous phase were disrupted, and the dispersed phase fused. The disappearance of the small droplet interface and the formation of large droplets led to aggregation and ultimately to the complete separation of the oil and aqueous phases. It has been shown that protein and oil droplets in almond milk flocculate and form oil–protein clusters. The aggregation of oil–protein clusters facilitated the phase separation process [25]. 

The microstructure of lipid droplets of flaxseed milk was illustrated via CLSM (Figure 2B). The fresh flaxseed milk (day 0) had lipid droplets (red) uniformly distributed throughout the aqueous phase (black). The lipid droplet particles had moved closer together by 150 days in storage at 25 °C. After 206 days in storage, the particle size of the lipid droplets increased, which could have been due to flocculation and agglomeration. In the flaxseed milk stored at 37 °C for 150 days, it was clearly observed that the small lipid droplet particles had aggregated together, but there was no significant change in particle size (Figure 1) at this time. This was presumably due to different measurement methods. Measurement of particle size required multiple dilutions, and if oil separation had occurred, the measured size of particles retained in the emulsion was small [26]. Then, large clusters of lipid droplets were observed. After 199 days, the previously tightly bound lipid droplets were observed to disperse, which was probably due to phase separation. 

The cryo-SEM results of flaxseed milk (Figure 2C) revealed the presence of a well-arranged three-dimensional network structure. The flaxseed gum and flaxseed protein entangled in the oil droplets were linked to the flaxseed membrane layer structure, resulting in a stable network structure. After 206 days at 25 °C and 150 days at 37 °C, the flaxseed gum filaments between the membrane layer structures showed significant fractures, and the shape of the oil body changed from smooth and full to uneven. However, the aggregation of flaxseed protein or oil bodies on the lamellar structure might have been due to the precipitation of oil from the oil bodies. After 206 days in storage at 37 °C, more small pores appeared in the lamellar structure of the flaxseed milk, and the adhesion between the lamellar structure and the oil body was reduced, which could also serve as an explanation for the fact that the originally stable large particle complex had broken down into smaller particles. The overall morphology of the lamellar structure would not change significantly during storage. In whole peanut milk, increased exposure of peanut proteins to hydrophobic groups improved water-holding and oil-mixing capacity. Peanut proteins were adsorbed on the surface of oil droplets to form complexes which formed a dense three-dimensional network structure and improved physical stability [27].

### 3.3. Viscosity

**Figure 3:**
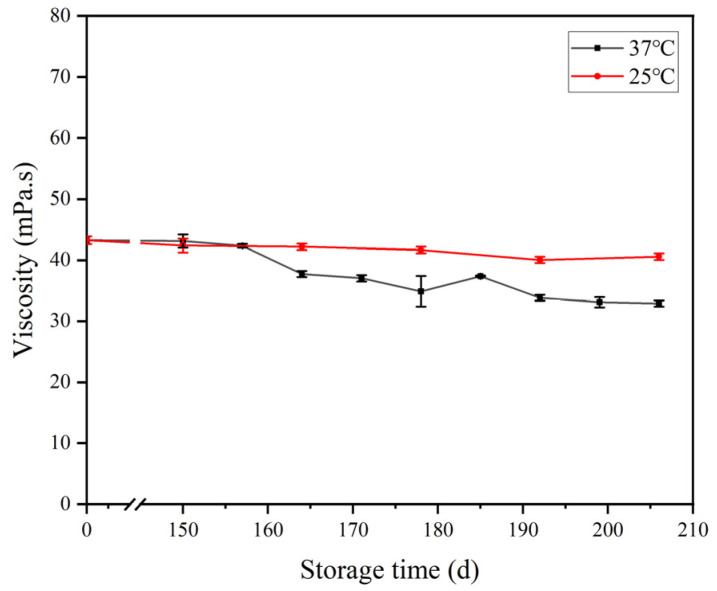
The viscosity of flaxseed milk stored at different temperatures.

Viscosity was one of the most important factors affecting the stability of flaxseed plant-based milk. The viscosity of the flaxseed milk during storage is shown in Figure 3. The initial viscosity of flaxseed milk was 43.25 mPa∙s. Studies have reported that the viscosities of commercial coconut milk and almond milk are 47.80 and 26.23 mPa∙s, respectively [10]. Unlike these commercial plant-based milk substitutes, the high viscosity of the flaxseed milk in this study was caused by flaxseed gum and protein. The high viscosity of the flaxseed milk led to a high surface tension, which effectively impeded the movement of suspended particles. It largely prevented collisions and agglomeration in the flaxseed milk and slowed down the settling of flaxseed milk, enhancing system stability [9]. The viscosity of the flaxseed milk stored at 25 °C for 206 days remained essentially unchanged. The viscosity of the flaxseed milk stored at 37 °C tended to decrease after 157 days. Subsequently, the viscosity of the flaxseed milk kept decreasing slowly. This might have been due to the disruption of the three-dimensional reticulation in the flaxseed milk over the increasing storage time. This also indicates that storage temperature has a strong influence on the viscosity of flaxseed milk, but without a significant effect at room temperature.

### 3.4. Gravity Separation Stability

**Figure 4:**
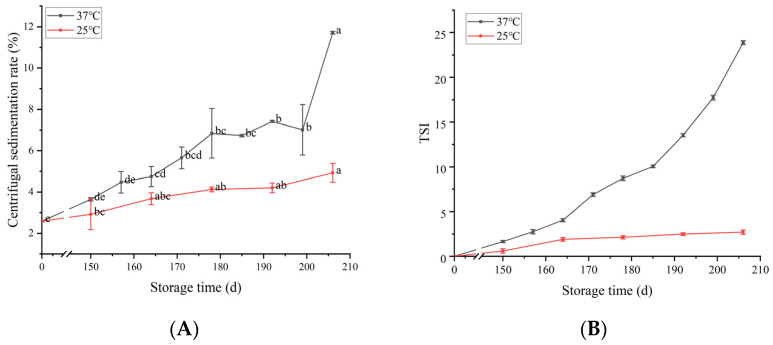
Centrifugal sedimentation rate (**A**) and TSI (**B**) of flaxseed milk stored at different temperatures. Different letters following the mean in the same column indicate significant differences (*p* < 0.05).

The stability of flaxseed milk was further characterized via monitoring of the centrifugal sedimentation rate and TSI during storage (Figure 4). Generally, centrifugal precipitation and TSI increased progressively during the storage period, which indicated a corresponding decrease in flaxseed milk stability. The centrifugal sedimentation rate and TSI values of the flaxseed milk stored at 25 °C increased slightly with increasing storage time. The centrifugal sedimentation rate and TSI growth rate of the flaxseed milk began to increase at 164 days in storage at 37 °C. At this point, the average particle size of flaxseed milk was 9.34 μm, and the presence of flocculation led to an increase in particle size. The flaxseed milk was an O/W emulsion, which is a thermodynamically unstable system. The large positive free energy at the interface of its oil droplets drove the droplets to coalesce. According to Stokes’ law, the larger the radius of the droplet, the greater the difference in density between the two phases and the faster the delamination or settling rate [28]. As storage time increased, the suspended particles in the flaxseed milk proceeded in Brownian motion, and aggregation occurred as particles collided with each other. The large particles were precipitated via gravity, which caused an increase in the centrifugal sedimentation rate of the flaxseed milk and an increase in the TSI coefficient.

### 3.5. Oxidative Stability

**Figure 5:**
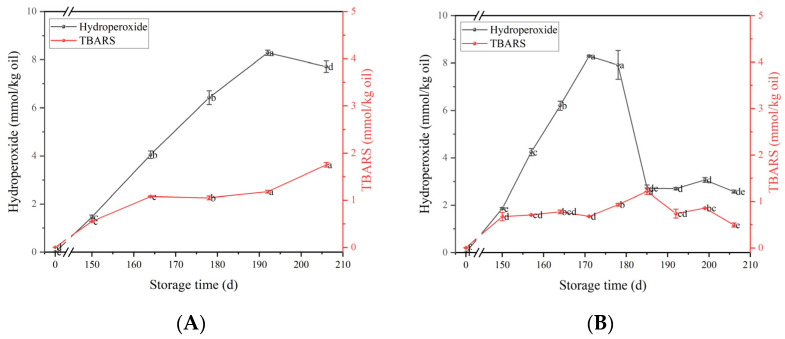
The primary and secondary products of flaxseed milk stored at 25 °C (**A**) and 37 °C (**B**). Different letters following the mean in the same column indicate significant differences (*p* < 0.05).

The concentration of primary (hydroperoxide) and secondary (TBARS) lipid oxidation products was monitored during storage to assess the oxidative stability of flaxseed milk. As shown in Figure 5A, the hydroperoxide concentration was highest at 8.29 mmol/kg oil during storage at 25 °C for 192 days. Figure 5B shows that the concentration of hydroperoxides of flaxseed milk stored at 37 °C initially increased with time, peaking at 171 days, and then decreased. The increase in TBARS concentration was rapid for 150 days and then decreased gradually over the next 60 days. The relative effect of different temperatures on the rate of lipid oxidation was analyzed via comparison of the linear slopes of the curves. The rate of lipid oxidation in the flaxseed milk stored at 37 °C was significantly higher compared to that of the milk stored at 25 °C. Hydroperoxide concentrations were maximal for the 25 °C milk at 192 days, with similar levels in the 37 °C milk at 171 days, which was probably their oxidative inflection point at this time. The absolute value of the potential was highest at the lowest hydroperoxide content for flaxseed milk stored at 37 °C for 171 days. It might be that the aggregation of large particles weakened the electrostatic interaction between the particles, thus leading to the change in potential, which also indicated a close relationship between physical and oxidative stability. Many studies have also demonstrated that remaining lipid oxidation products are generally captured at the interface and adversely affect the physical stability of the emulsion [24]. During storage, the level of hydroperoxides produced increased for 171 days and then decreased over a longer storage period. This might be due to the fact that the rate of decomposition of the primary reaction products was faster than their formation over long storage times. At the same time, TBARS levels steadily increased to a maximum and then decreased over a longer storage time. This indicated that further degradation led to the formation of volatile products, which would cause products to sour, which is unacceptable to consumers. 

### 3.6. The Influence of Polyphenol Partitioning on Oxidative Stability of Flaxseed Plant-Based Milk

**Figure 6:**
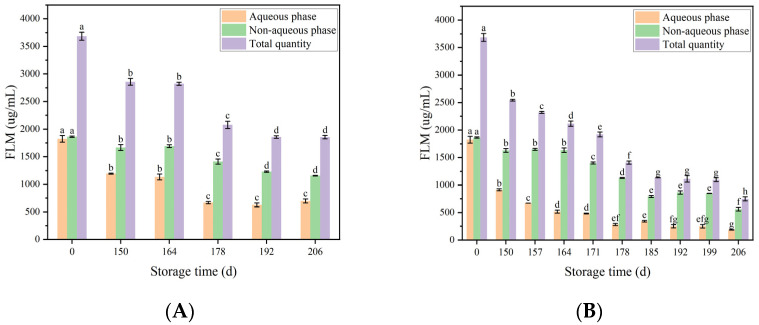
Flaxseed lignans migration in aqueous and non-aqueous phases of flaxseed milk at 25 °C (**A**) 37 °C (**B**). Different letters following the mean in the same column indicate significant differences (*p* < 0.05).

Flaxseed lignans are phenolic compounds, which mainly present in polymer complexes in flaxseed. Studies have demonstrated the antioxidant effect of flaxseed lignans in flaxseed oil emulsion [15,29]. The distribution of the flaxseed lignan macromolecules (FLM) in flaxseed milk is shown in Figure 6. Initially, the flaxseed lignans were distributed evenly in the oil–water phase of the flaxseed milk. Overall, the flaxseed lignin content all decreased with increasing storage time. However, it was obvious that the rate of reduction in lignin content in the aqueous phase of the flaxseed milk stored at both temperatures was greater than that in the non-aqueous phase. This might be due to partial depletion or migration of FLM in the aqueous phase to the oil phase or to particle surfaces. Previous studies have calculated the topological polar surface area (TPSA) of different flaxseed lignins, which suggests potential penetration into the interior of lipid droplets due to the lower TPSA values of flaxseed lignans [30]. That was why the flaxseed lignin content in the non-aqueous phase remained essentially constant between 150 and 164 days in storage. In addition, the number and position of phenolic hydroxyl groups and hydrophobic groups, such as phenylmethyl, played an important role in the stability and antioxidant activity of phenolic compounds in O/W emulsions [31,32]. Interestingly, the flaxseed lignin content in the non-aqueous phase also started to decrease in the flaxseed milk stored at 37 °C at 171 days. Corresponding to what has been mentioned above, oiling-off in the upper layer of flaxseed milk could be clearly observed and the content of hydroperoxide values peaked at this time. This might be explained by the leakage of lignin from the non-aqueous phase due to the breaking of flaxseed milk, which was heavily depleted for antioxidant purposes. This also showed that the relationship between chemical oxidation and physical destabilisation of flaxseed milk was inextricably linked.

### 3.7. Statistical Correlation Analysis

**Figure 7:**
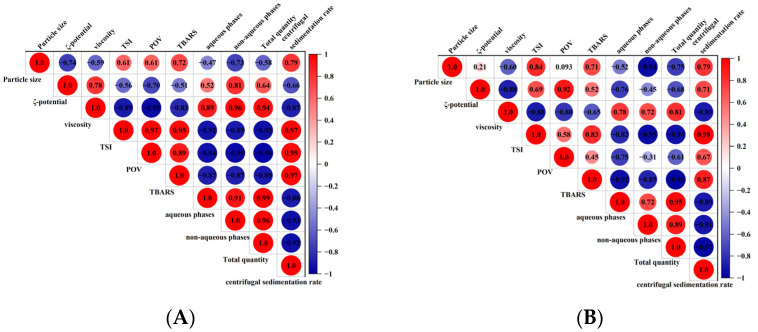
Results of statistical correlation analysis of the physical and chemical stability of flaxseed milk in storage at 25 °C (**A**) 37 °C (**B**).

The results of the statistical correlation analysis of the physical and chemical stability of the flaxseed milk are shown in Figure 7. The particle size of the flaxseed milk was positively correlated with the centrifugal sedimentation rate, TSI coefficient, POV, and TBARS content and negatively correlated with the flaxseed lignin content. These results suggest that the chemical oxidation of flaxseed milk is closely related to physical stability. The larger the particle size, the more unstable the whole system, which was also consistent with Stokes’ law. Furthermore, the concentrations of POV and TBARS were negatively correlated with the flaxseed lignin content of each phase and the total lignin content. This also suggests that flaxseed lignans have antioxidant properties for flaxseed milk. There was some variation in the effect and correlation of physical stability and chemical oxidation rates when stored at different temperatures. Physical destabilization and chemical oxidation reactions in flaxseed milk stored at 37 °C were more intense than those stored at 25 °C.

## 4. Conclusions

We studied the storage stability of flaxseed milk at different temperatures. It showed that chemical oxidation was closely linked to physical destabilization. The values of particle size, TSI, and centrifugal sedimentation rate of flaxseed milk gradually increased with increasing storage time, which indicated a decrease in physical stability. At the same time, the change in the potential of the flaxseed milk coincided with a change in the hydroperoxide content, showing that the change in physical stability was accompanied by a drastic oxidative change. The flaxseed lignans in the flaxseed milk were resistant to oxidation, and the degree of influence depended on the physical location and content of the flaxseed lignans. The degree of oxidation was also proven to affect the microstructure of the flaxseed milk, by causing stabilization–destabilization the flaxseed milk interface to collapse. The study was important for exploring the stabilization–destabilization mechanism of flaxseed milk. It would also hopefully be useful in elucidating the changes in storage stability of other plant-based milk substitutes.

## Data Availability

The data used to support the findings of this study can be made available by the corresponding author upon request.
